# Renal biopsies performed before versus during ablation of T1 renal tumors: implications for prevention of overtreatment and follow-up

**DOI:** 10.1007/s00261-020-02613-4

**Published:** 2020-06-20

**Authors:** Christiaan V. Widdershoven, Brigitte M. Aarts, Patricia J. Zondervan, Michaël M. E. L. Henderickx, Elisabeth G. Klompenhouwer, Otto M. van Delden, Warner Prevoo, Alexander D. Montauban van Swijndregt, Reindert J. A. van Moorselaar, Axel Bex, Brunolf W. Lagerveld

**Affiliations:** 1 Department of Urology, Amsterdam University Medical Centers, Meibergdreef 9, 1105 AZ Amsterdam, The Netherlands; 2 Department of Radiology, The Netherlands Cancer Institute, Plesmanlaan 121, 1066 CX Amsterdam, The Netherlands; 3 GROW School for Oncology and Developmental Biology, Maastricht University Medical Centre, P.O. Box 5800, 6202 AZ Maastricht, The Netherlands; 4 Department of Radiology, Amsterdam University Medical Centers, Meibergdreef 9, 1105 AZ Amsterdam, The Netherlands; 5 Department of Radiology, OLVG, Oosterpark 9, 1091 AC Amsterdam, The Netherlands; 6 Department of Urology, The Netherlands Cancer Institute, Plesmanlaan 121, 1066 CX Amsterdam, The Netherlands; 7 Specialist Centre for Kidney Cancer, Royal Free London NHS Foundation Trust and UCL Division of Surgery and Interventional Science, Pond Street, London, NW3 2QG UK; 8 Department of Urology, OLVG, Oosterpark 9, 1091 AC Amsterdam, The Netherlands

**Keywords:** Renal mass biopsy, Renal, Kidney, Thermal ablation

## Abstract

**Purpose:**

To assess the difference between renal mass biopsy (RMB) performed either before or during the ablation procedure.

**Methods:**

A retrospective multicenter study was performed in patients with a cT1 renal mass treated with ablation between January 2007 and July 2019, including a search in the national pathology database for patients with a RMB planned for ablation. Patient and tumor characteristics and information on malignant, benign, and non-diagnostic biopsy results were collected to establish rates of overtreatment and number of ablations avoided in case of benign or non-diagnostic histology.

**Results:**

RMB was performed in 714 patients, of which 231 patients received biopsy before planned ablation, and 483 patients at the time of ablation. Pathology results before ablation were malignant in 63% (145/231), benign in 20% (46/231) and non-diagnostic in 17% (40/231). Pathology results at the time of ablation were malignant in 67.5% (326/483), benign in 16.8% (81/483) and non-diagnostic in 15.7% (76/483), leading to a total of 32.5% of ablation of benign or non-diagnostic lesions. Of the patients with a benign biopsy obtained before ablation, 80.4% (37/46) chose not to undergo ablation. Patients with inconclusive biopsy before planned ablation chose an informed individualized approach including ablation, repeated biopsy, or no intervention in 56%, 34% and 10%.

**Conclusion:**

This study emphasizes the importance of obtaining a biopsy prior to the ablation procedure in a separate session to lower the rate of potentially unnecessary ablations.

## Introduction

Renal cell carcinoma (RCC) accounts for 2–3% of all malignancies worldwide and is the tenth most common tumor in both males and females in western countries [1, 2]. The incidence of small localized RCC lesions (< 4 cm) has dramatically increased in the last decade due to the expansion of imaging modalities. Standard treatment for small renal masses (SRMs) is a partial nephrectomy [3], but in comorbid or elderly patients, a more minimal invasive treatment can be preferred, such as focal thermal ablation or active surveillance or stereotactic radiotherapy [4, 5].

Although some renal masses show malignant characteristics on radiological images, up to 30% are found to be benign after (partial) nephrectomy [6, 7]. Renal mass biopsy (RMB) can be performed to differentiate between a malignant or benign renal tumor. The renal cancer guideline of the European Association of Urology (EAU) recommends to perform a percutaneous RMB before the ablation procedure [4]. However, in clinical practice, different strategies are applied with biopsies performed either before the ablation in a separate session or at the time of the ablation procedure itself.

The reason for performing a biopsy at the time of ablation is regularly to allow both procedures to be performed in one session. The disadvantage of this strategy is overtreatment or uncertainty regarding follow-up in case of pathological assessment or remains non-diagnostic [8, 9].

This study aims to evaluate the differences between RMB performed before or at the time of ablation in terms of histological diagnosis and rate of treatment of benign tumors and uncertainty.

## Methods

### Patient selection

This multicenter retrospective study was conducted in the Renal Cancer Network Amsterdam, consisting of the OLVG hospital, the Netherlands Cancer Institute (NKI), and Amsterdam University Medical Centers (AUMC). The study had appropriate institutional review board approval from each institution. A data transfer agreement was obtained to execute a pooled data analyses of the three centers together.

Patients treated by ablation for a renal tumor between January 2007 and July 2019 were included. Furthermore, the PALGA database, the nationwide network and registry of histo- and cytopathology in the Netherlands, was searched for patients who were diagnosed by means of an RMB in the three centers mentioned above and were not treated due to either a benign or non-diagnostic biopsy result. All patients were discussed in a multidisciplinary tumor board consisting of urologists, medical oncologists and (interventional) radiologists and these reports were retrospectively evaluated. If the board decided that the patient was not intended to be treated with an ablation, the patient was excluded from this study. Consequently, patients planned for (partial) nephrectomy or with a renal infection or abscess were excluded. If the board advised a patient to be treated with an ablation, the patient would be included.

Only primary tumors limited to the kidney and smaller than 7 cm were included (cT1 tumors). Patients were excluded from the study in case of prior surgery or ablation of the kidney or intrarenal metastases from another primary malignancy.

Renal masses were diagnosed by a board of certified abdominal radiologists on multiphase computed tomography. An RCC was suspected when a hypervascular mass was found on contrast-enhanced computed—tomography (CT). Lesions were considered suspicious for malignancy when an increase of at least 15 Hounsfields units after contrast administration was observed. When a suspected lesion was found on US, a multiphase CT with an arterial, corticomedullary and nephrogenic phase was performed. From 2017 onwards, all patients (treated and non-treated) were discussed in a multicenter multidisciplinary tumor board between the three centers during a weekly video conference meeting. Patients with a tumor ≤ 4 cm (cT1a) were offered the choice between surgical and ablative procedures or active surveillance, unless patient comorbidity supported a specific choice. Ablation for tumors > 4 cm and ≤ 7 cm (cT1b) was only offered for highly comorbid patients who were not eligible for surgery. All procedures were performed or supervised by an experienced, board certified, interventional radiologist.

### Biopsy before the ablation procedure

Biopsies before the ablation were taken in a separate session from the ablation procedure. RMB was guided by ultrasound (US) or CT under local anesthesia. Different biopsy needles were used in each hospital (Center 1: Argon Medical 18G, Center 2: co-axial Bard® Magnum® 18G, Center 3: Cook 16 G or 18G). After the biopsy procedure, patients were given 2-h bed rest with monitoring of vital signs.

### Biopsy at the time of ablation

Biopsies were taken in the same session as the ablation procedure, immediately before the start of the thermal ablation. The ablation procedure was performed by US and/or (cone beam) CT guidance under local or general anesthesia depending on the patient, tumor characteristics and preference of the physician. Different ablation techniques, such as radio frequency ablation, micro wave ablation, laparoscopic and percutaneous cryoablation were used in the three hospitals.

### Pathologic evaluation

Histological assessment was performed at each hospital according to the EAU guidelines by an experienced genitourinary pathologist. The Union for International Cancer Control/American Joint Committee on Cancer classification was used when an RCC was diagnosed by the pathologist [4]. Results were regarded as diagnostic if the tissue sample was either defined as definitively benign or malignant or if a preference for a diagnosis was made by the pathologist. Biopsies were considered non-diagnostic if the biopsy tissue only contained normal parenchyma, fibrosis, and necrosis or was not representative. In case of an oncocytic neoplasma in which no differentiation between a benign or malignant oncocytic lesions could be performed, the lesion was classified as “no diagnosis” (Table 2). For the pathological analysis, the specific staining protocol for the suspicion of renal neoplasms was followed. Those stains included cytokeratins, vimentin, cadherins, RCC Marker, and other RCC-specific markers. The analyses were performed by a board certified urogenitary pathologist.

### Data analysis

**Fig. 1:**
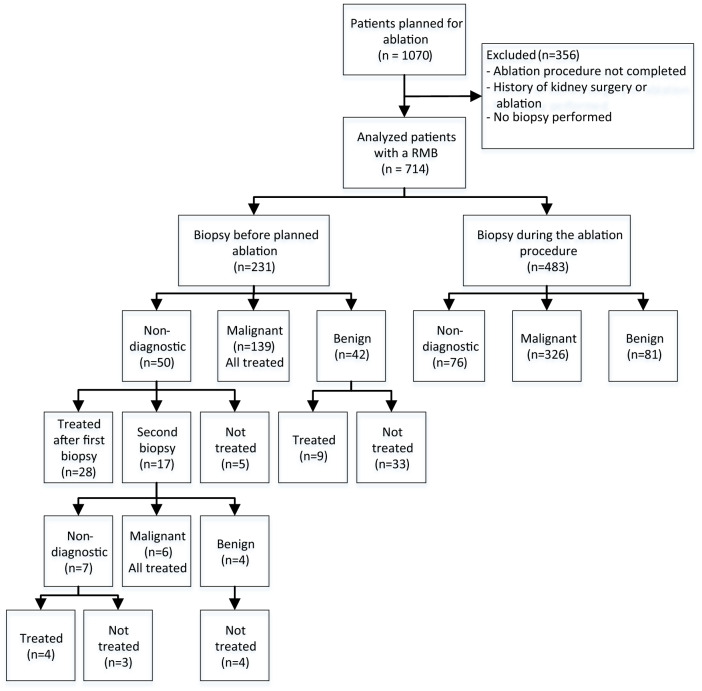
Flow chart of the biopsies performed before or at the ablation

Patient and radiologic tumor characteristics and pathology results were collected to establish rates of diagnostic accuracy, overtreatment, and avoided ablation in case of benign histology. Patients were divided into two groups: those receiving a biopsy before a planned ablation, and those receiving a biopsy during the ablation procedure (see Fig. 1). Adverse events after the biopsy were classified according to the Clavien–Dindo classification [10].

Continuous variables are shown as median with interquartile range and categorical variables as numbers and percentages. Differences between groups were calculated by the Chi-square method for the categorical data and an independent *t* test and Mann–Whitney U test for the numerical variables. All tests were two-sided and p-values below 0.05 were considered statistically significant. Statistical analysis was conducted with SPSS V.25 (IBM Corp, Armonk, NY, USA).

## Results

### Pretreatment characteristics

A total of 1070 patients were scheduled for thermal ablation in the three hospitals between January 2007 and July 2019. Of these, 356 patients did not meet the inclusion criteria and were excluded, in the majority of the cases because they received treatment without a biopsy, multiple ablations in the same patient (whereby only the first ablation was taken into account), treatment of tissue other than the kidney or prior history of renal surgery, leaving 714 patients for analysis (Fig. 1 and Table 1).

**Table 1:** Patient characteristics

Patient characteristics	Total group (*n *= 714)	Biopsy performed in a separate session before the ablation procedure (*n *= 231)	Biopsy performed in same session as the ablation procedure (*n *= 483)	*p*
Median age *(IQR)*	69 (61–75)	68 (59–74)	70 (62–76)	0.070
Gender				
Male. *n (%)*	448 (62.7%)	152 (65.8%)	296 (61.3%)	0.250
Female. *n (%)*	266 (37.3%)	79 (34.2%)	187 (38.7%)	0.250

Significance was assumed for *p *< 0.05

### Biopsy before planned ablation in a separate session

231 patients underwent a biopsy in a separate session before the ablation. Pathology results after the first biopsy were malignant in 60.2% (139/231), benign in 18.2% (42/231), and non-diagnostic in 21.6% (50/231).

In the benign group, 33/42 patients (78.5%) were not treated as a result of the benign pathology result. Nine patients in this group wished to be treated. Of the 50 patients in the non-diagnostic group, 28 chose ablation. In 17 patients, a second biopsy was performed, resulting in six malignant biopsies (all treated), four benign biopsies (all not treated), and seven non-diagnostic biopsies (three treated). Altogether, a total of 37 patients (80% of the benign tumors) chose not to undergo a planned ablation, due to a benign biopsy result.

After the second biopsy, the final pathology results before ablation were malignant in 63% (145/231), benign in 20% (46/231), and non-diagnostic in 17% (40/231). In the group of 40 patients who had a non-diagnostic result after a second biopsy, 32 opted for treatment, and 8 were not treated with an ablation for personal reasons (see Fig. 1). Histological results of these biopsies are shown in Table 2.

**Table 2:** Pathology results after 1–2 biopsies before treatment and 1 biopsy during treatment

Pathology result	Total group (*n *= 714)	Biopsy performed in a separate session before the ablation procedure (*n *= 231)	Biopsy performed in same session as the ablation procedure (*n *= 483)
*Malignant*	471 (66.0%)	145 (62.7%)	326 (67.5%)
B-cell lymphoma	1 (0.1%)	0 (0.0%)	1 (0.2%)
Chromophobe	27 (3.8%)	6 (2.6%)	21 (4.3%)
Clear cell	337 (47.2%)	93 (40.3%)	244 (50.5%)
Eosinophile	2 (0.3%)	0 (0.0%)	2 (0.4%)
NOS	18 (2.5%)	12 (5.2%)	6 (1.2%)
Papillary	28 (3.9%)	15 (6.5%)	13 (2.7%)
Papillary type 1	44 (6.2%)	14 (6.1%)	30 (6.2%)
Papillary type 2	13 (1.8%)	5 (2.2%)	8 (1.7%)
Papillary type 1 + 2	1 (0.1%)	0 (0.0%)	1 (0.2%)
*Benign*	127 (17.8%)	46 (14.6%)	81 (16.8%)
Angiomyolipoma	24 (3.4%)	10 (4.3%)	14 (2.9%)
Leiomyoma	2 (0.3%)	1 (0.4%)	1 (0.2%)
Oncocytoma	100 (14.0%)	34 (14.7%)	66 (13.7%)
Xanthogranuloma	1 (0.1%)	1 (0.7%)	0 (0.0%)
*Non*-*diagnostic*	116 (16.2%)	40 (17.3%)	76 (15.7%)
No diagnosis	28 (3.9%)	17 (7.4%)	11 (2.3%)
Of which ‘oncocytic’	6 (21.4%)	2 (11.7%)	4 (36.3%)
Not representative	38 (5.3%)	8 (3.5%)	30 (6.2%)
Normal kidney parenchyma	30 (4.2%)	5 (2.2%)	25 (5.2%)
Reactive	20 (2.8%)	10 (4.3%)	10 (2.1%)

### Biopsy during the ablation procedure

In 483 patients, a biopsy was only performed during the ablation procedure, without having information about the histology of the tumor prior to ablation. The decision to perform an ablation was based upon high suspicion of malignancy on imaging. In this group, 81 (16.8%) benign tumors were treated, 76 (15.7%) tumors with a non-diagnostic pathology result and 326 (67.5%) malignant tumors. Together, 157 patients (32.5%) with a proven benign or non-diagnostic lesion were treated.

### Potential overtreatment

In the group receiving a biopsy before a planned ablation, 80.5% of the biopsies resulted in an ablation procedure, compared to 100% in the group receiving only a biopsy during ablation. In the group only receiving a biopsy during the ablation procedure, overtreatment might have occurred in 32.5% of all ablations due to a benign or non-diagnostic biopsy result, compared to 17.7% in the group undergoing a biopsy before planned ablation and who chose for ablation despite being informed about the result (*p *< 0.001). There were no significant differences in age, gender, or tumor size between the groups of patients who received a biopsy in a separate session before the ablation and the group who received a biopsy at the time of ablation.

### Adverse events

Adverse events related to the renal biopsy were reported in 3 of the 714 patients (0.4%), all of which occurred in the patients who underwent the biopsy in a separate session before the ablation. One patient (0.14%) developed a grade 3a adverse event after a biopsy before ablation (histological result: oncocytoma) consisting of a false aneurysm which was treated with embolization. The other two adverse events were grade 1, both being patients who did not feel well after the procedure and had to be observed in the hospital, with no further medical or interventional measures taken.

## Discussion

Percutaneous biopsies performed in experienced centers have a high diagnostic yield, specificity and sensitivity, and low morbidity [11]. In this multicenter study, we evaluated the implications for treatment decisions and follow-up of biopsies performed before ablation in a separate session or during ablation. We showed that performing a biopsy during the ablation procedure led to 16.8% benign and 15.7% non-diagnostic biopsies, suggesting a potentially unnecessary ablation of renal masses in 32.5%. In addition, 19.5% of the patients who were diagnosed with a benign or non-diagnostic biopsy result decided not to undergo their planned ablation. A total of 37 patients (80% of the benign tumors) chose not to undergo a planned ablation, due to a benign biopsy result. Performing a biopsy before ablation therefore reduces overtreatment and allows patients and physicians to adjust clinical management according to the biopsy results.

In this cohort, biopsies performed during the ablation procedure led to an ablation of a histologically confirmed benign tumor in 16.8% of the patients. Although that is just marginally higher than previous reports of ablation series (ranging from 2 to 14%), it is in line with the rates reported after surgery of small renal masses without previous biopsy (up to 30%) [7, 12, 13]. Results from our retrospective analysis demonstrate that patients who were involved in the choice of management after a benign biopsy result chose to not undergo the ablation in the majority of the cases (80%) and therefore avoided an unnecessary treatment.

Performing a biopsy during the ablation procedure eliminates the possibility to perform an additional biopsy and creates uncertainty for follow-up in case of an inconclusive biopsy. In our cohort, 15.7% of the patients who received a biopsy in the same session as the ablation procedure had a non-diagnostic biopsy. Physicians therefore had to consider that the renal mass was malignant and followed an intensive follow-up scheme of multiple CT or MRI scans. Contrast-enhanced imaging, necessary for detection of recurrence after ablation, can be a burden in patients with chronic kidney disease including most patients planned for ablation and, in case of CT scans, expose patients to radiation. In case a non-diagnostic biopsy could be repeated and confirmed as benign, a less intensive follow-up scheme of active surveillance would have been followed. In the literature, this is reported in up to 33% of the beforehand suspected malignant renal masses after partial nephrectomy [8, 9].

Taking a renal mass biopsy (RMB) in a separate session has additional advantages. First, Tsang Mui Chung et al. suggested that for percutaneous image guided ablations, an RMB can inform the interventional radiologist about the actual ablation procedure regarding patient position and percutaneous approach in case the same image guided modality is used [14]. Second, clinical impact can be achieved according to histologic subtype before treatment.

On the other hand, performance of a biopsy during the ablation avoids the need for another hospital visit. Several patients chose to undergo an ablation regardless of the benign histological result, in our cohort, 19.5%. Reasons for this were, for example, that patients still preferred a treatment and, in case of an angiomyolipoma, to lower the risk for bleeding [15, 16]. Also, the risk of a biopsy can be substantial in high risk patients on anticoagulation that cannot be paused. In our cohort, only one biopsy required an intervention. Physicians need to discuss the different histological results and their consequences with the patient in a shared decision process including the need for a biopsy.

In literature, only two other groups investigated the timing of the biopsy in patients undergoing ablation. Wells et al. recommend to perform a biopsy before the ablation in a separate session with the opportunity to take a second biopsy during the ablation. However, they only included patients who underwent ablation and consequently did not report changes of clinical management due to a benign biopsy before treatment [6]. Tsang Mui Chung et al. concluded that a biopsy during the ablation did not lead to overtreatment, because only 3% of the ablated lesions were benign. However, they included oncocytoma as not definitively benign in their analysis, which consequently lowered the rate of benign renal masses [13]. Differentiation between oncocytomas and low grade oncocytic neoplasms is difficult, since, as reported in literature, up to 35% of the oncocytomas by means of biopsy receives a different histological diagnosis after surgical resection [17]. In the present study, we therefore classified oncocytic lesions that were not strictly marked as an oncocytoma as “no diagnosis,” instead of an oncocytoma, and only used oncocytoma when this was strictly classified as such by the pathologist. In our study, we found 6 cases where the pathologist could not further specify the oncocytic lesion, and we have therefore included those in the “no diagnosis” group.

As with all retrospective studies, one of the main limitations of our study is that it is subject to bias, in particular selection bias. In this study, no randomization was performed which resulted in a relatively small number of biopsies performed before the ablation in a separate session. Our results, however, seem to contradict this management and propagate performing the biopsy before planned ablation to avoid unnecessary treatment of benign lesions and uncertainty of follow-up of inconclusive lesions.

## Conclusion

This study emphasizes the importance of obtaining a biopsy prior to the ablation procedure in a separate session to lower the rate of unnecessary ablations.

## Abbreviations

RCC: Renal cell carcinoma
SRM: Small renal mass
RMB: Renal mass biopsy
EAU: European Association of Urology
US: Ultrasound
CT: Computed—tomography
MRI: Magnetic resonance imaging
